# Exosomes derived from microRNA-512-5p-transfected bone mesenchymal stem cells inhibit glioblastoma progression by targeting JAG1

**DOI:** 10.18632/aging.202747

**Published:** 2021-03-26

**Authors:** Tengfeng Yan, Miaojing Wu, Shigang Lv, Qing Hu, Wenhua Xu, Ailiang Zeng, Kai Huang, Xingen Zhu

**Affiliations:** 1 Department of Neurosurgery, The Second Affiliated Hospital, Nanchang University, Nanchang, P.R. China; 2 Department of Neurosurgery, Jiujiang No.1 People’s Hospital, Jiujiang, P.R. China; 3 Department of Neurosurgery, The First Affiliated Hospital, Nanjing Medical University, Nanjing, P.R. China

**Keywords:** bone mesenchymal stem cell (BMSC), exosomes, miR-512-5p, jagged 1 (JAG1), glioblastoma (GBM)

## Abstract

In this study, we demonstrate that bone mesenchymal stem cell (BMSC)-derived exosomes alter tumor phenotypes by delivering miR-512-5p. miR-512-5p was downregulated in glioblastoma tissues and cells, and Jagged 1 (JAG1) was the target gene of miR-512-5p. We clarified the expression patterns of miR-512-5p and JAG1 along with their interactions in glioblastoma. Additionally, we observed that BMSC-derived exosomes could contain and transport miR-512-5p to glioblastoma cells *in vitro*. BMSC-derived exosomal miR-512-5p inhibited glioblastoma cell proliferation and induced cell cycle arrest by suppressing JAG1 expression. *In vivo* assays validated the *in vitro* findings, with BMSC-exosomal miR-512-5p inhibiting glioblastoma growth and prolonging survival in mice. These results suggest that BMSC-derived exosomes transport miR-512-5p into glioblastoma and slow its progression by targeting JAG1. This study reveals a new molecular mechanism for glioblastoma treatment and validates miRNA packaging into exosomes for glioblastoma cell communication.

## INTRODUCTION

Glioblastoma (GBM) is the most common primary malignant brain tumor in adults [1]. Gliomas are classified by the World Health Organization (WHO) grades of I-IV based on their histology and malignant behavior. GBM is the most aggressive form, and the overall survival (OS) for patients is relatively short (less than 1–1.5 years) [2, 3]. GBM treatment options include surgical resection, radiotherapy, and combined temozolomide (TMZ) chemotherapy. However, the clinical efficacy of these approaches is not great [4]. Therefore, to find more effective targets for GBM therapies, it is necessary to explore novel mechanisms of GBM progression.

MicroRNAs (miRNAs) can achieve physiological or pathological effects by binding to the 3'-untranslated region (3'-UTR) or open reading frames (ORFs) of their target mRNAs [5]. Almost 2588 mature miRNAs have been identified in humans. A number of miRNAs are expressed differentially in benign and malignant tumors, including GBM [6, 7]. The regulatory functions of miRNAs play a critical role in GBM by affecting cell proliferation [8], apoptosis [9], drug resistance [10], and metastasis [11]. Our previous studies indicated that several miRNAs could mediate GBM progression [12–15]. miR-512-5p was recently reported as an anti-oncogene in non-small cell lung carcinoma [16] and gastric carcinoma [17]. However, the expression of miR-512-5p and its potential role in GBM progression are not known. In our study, the downregulation of miR-512-5p in GBM tissues and cells provided important clues on the alteration of GBM progression. The underlying mechanism of miR-512-5p in GBM progression was further investigated both *in vitro* and *in vivo*.

JAG1 is a cell surface ligand that primarily acts through the highly conserved Notch signal pathway, which influences tumor development [18, 19]. JAG1 was first discovered in breast cancer patients, and high JAG1 expression implied significantly shorter overall survival [20]. Some research indicated that miRNAs mediate the Notch signal pathway to regulate tumor cell invasion [21], epithelial-mesenchymal transition (EMT) [22], and treatment resistance [23, 24]. Moreover, the upregulation of JAG1 can promote the malignant progression of glioma [25, 26]. Exosomes are a subtype of extracellular vesicle (EV) with a lipid bilayer ranging from 30–150 nm. They are naturally released by all cell types, and shed into various biological fluids such as urine, plasma, and serum [27]. Recent studies reported that exosomes could transfer miRNAs to recipient cells, which can alter their biological function [28]. Bone mesenchymal stem cells (BMSCs) can home to tumor areas after intravenous injection and engineered BMSCs may be effective vehicles for delivering therapeutic agents to glioma. Interestingly, current evidence suggests that exosomes of BMSCs are an effective treatment for glioma [29]. Based on the previous studies, we hypothesized that BMSC exosomes could deliver miR-512-5p to GBM and investigated the potential mechanism.

## RESULTS

### miR-512-5p is poorly expressed in GBM specimens and cell lines

**Figure 1:**
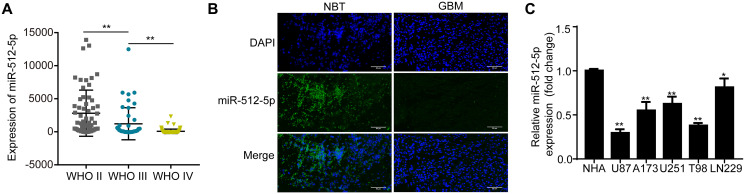
**miR-512-5p is poorly expressed in GBM specimens and cell lines**. (**A**) miR-512-5p expression in glioma tissues from the CCGA database as determined by RT-qPCR. (^**^p < 0.01, Student's *t*-test) (**B**) miR-512-5p expression in non-tumor brain tissues (NBTs) and GBM tissues as detected by FISH (200 ×). (**C**) miR-512-5p expression in five GBM and NHA cell lines as determined by RT-qPCR. (^*^p < 0.05, ^**^p < 0.01, one-way ANOVA, compared NHA with GBM cell lines). Data are represented as the mean ± standard deviation of three independent experiments.

The miRNA expression dataset of glioma was obtained from the Chinese Glioma Genome Atlas (CGGA) database (*n* = 198). According to the WHO Classification of glioma, the level of miR-512-5p in different grades of glioma was negatively correlated with the degree of malignancy (Figure 1A). The miR-512-5p levels in glioma tissues (WHO I, *n* = 7; WHO II, *n* = 26; WHO III, *n* = 10; WHO IV, *n* = 28) and non-tumor brain tissues (NBT, *n* = 8) were determined by RT-qPCR and FISH. miR-512-5p expression was significantly lower in high-grade glioma (HGG) tissues compared with low-grade glioma (LGG) tissues (Table 1, *p* = 0.0027). Moreover, FISH assays revealed that miR-512-5p expression was lower in GBM tissues compared to NBT (Figure 1B). We also measured miR-512-5p expression in GBM cell lines (U87, U251, A173, T98, LN229) and a NHA cell line by RT-qPCR, and found that miR-512-5p expression was downregulated in the GBM cell lines (Figure 1C). Taken together, our results indicate that miR-512-5p has decreased expression in GBM.

**Table 1:** Correlation between the clinicopathologic characteristics and miR-512-5p expression in glioma (*n* = 71).

**Variables**	***n***	**miR-512-5p expression**		***P*-Value**
		**low (0&1)**	**high (2&3)**	
Ages				0.1081
≤ 50	30	13	17	
> 50	41	15	26	
Genders				0.488
Female	27	10	17	
Males	44	18	26	
Tumor differentiation				0.0027
I/II	33	6	27	
III/IV	38	22	16	

### Overexpression of miR-512-5p inhibits GBM cell proliferation and causes cell cycle arrest

**Figure 2:**
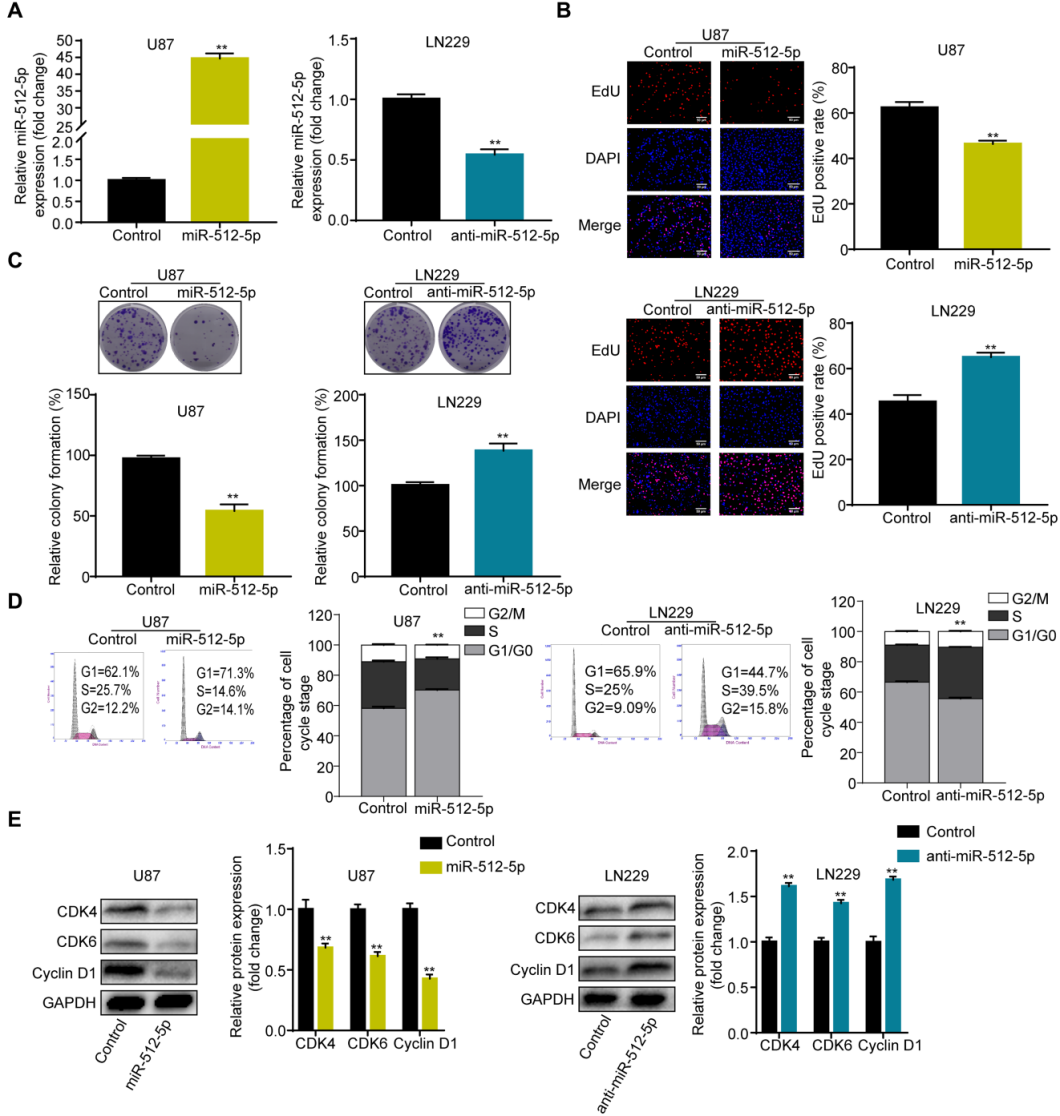
**Overexpression of miR-512-5p inhibits GBM cell proliferation and causes cell cycle arrest.** (**A**) miR-512-5p expression in U87 and LN229 cells determined after transfecting cells with corresponding vectors by RT-qPCR. (**B**) Cell proliferation activity as examined by EdU assays. (200 ×) (**C**) Cell proliferation activity as examined by colony formation assays. (**D**) Analysis of cell cycle by flow cytometry. (**E**) G1-arrest-relevant cell cycle regulators determined by western blotting. Data are represented as the mean ± standard deviation of three independent experiments. ^*^p < 0.05, ^**^p < 0.01, Student's *t*-test, compared to the Control group.

To explore the biological significance of miR-512-5p in GBM, we performed gain- and loss-of-function experiments in the U87 and LN229 cell lines. As shown in Figure 2A, the U87 and LN229 cell lines were successfully transfected with miR-512-5p and anti-miR-512-5p. Furthermore, we performed CCK-8, EdU, colony formation, and flow cytometry assays to evaluate the role of miR-512-5p in GBM cell proliferation and cell cycle. Upregulation of miR-512-5p expression significantly inhibited cell proliferation and cell cycle G1-S phase transition in U87 cells, whereas downregulation of miR-512-5p increased cell proliferation and cell cycle G1-S phase transition in LN229 cells (Figure 2B–2D). Western blotting analysis of G1-arrest-relevant cell cycle regulators confirmed the causes of miR-512-5p-mediated G1-S phase cell cycle arrest. Western blotting analysis also showed that cell cycle-regulated proteins including CDK4, CDK6, and Cyclin D1 were downregulated in U87 with miR-512-5p overexpression, while inhibition of miR-512-5p led to CDK4, CDK6, and Cyclin D1 upregulation (Figure 2E). These results propose that miR-512-5p overexpression inhibits cell proliferation and induces cell cycle arrest in GBM cells.

### miR-512-5p targets JAG1

**Figure 3:**
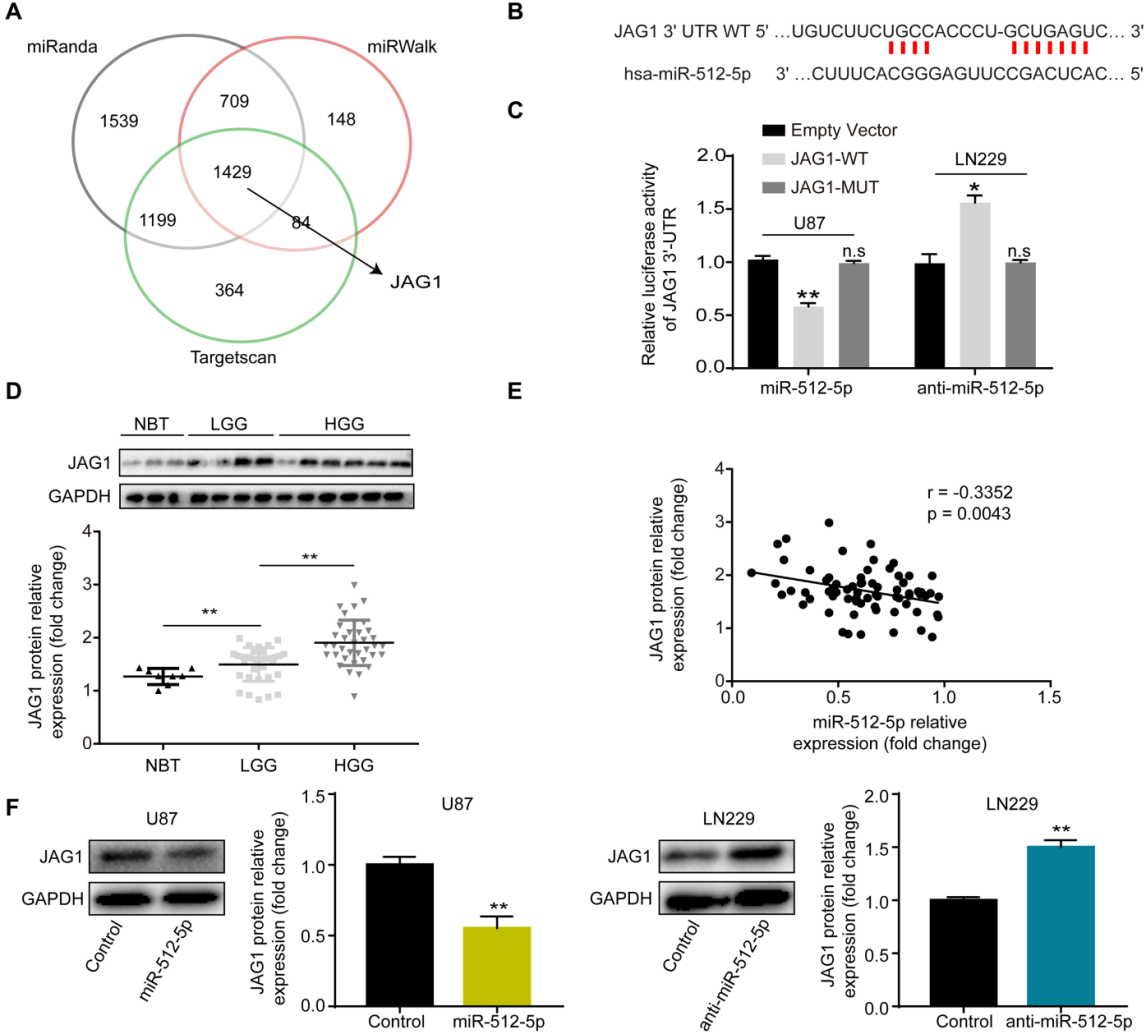
**miR-512-5p targets JAG1.** (**A**) Venn diagrams show the targets of miR-512-5p. (**B**) The predicted miR-512-5p binding site on JAG1 3'-UTR (wild type, WT) is shown in red. (**C**) Dual luciferase reporter assays were used to confirm the binding site of miR-512-5p and JAG1. ( n.s, no significance, ^*^p < 0.05, ^**^p < 0.01, Student's *t*-test, compared to the JAG1-WT group.) (**D**) JAG1 expression in NBTs (*n* = 8) and glioma tissues (*n* = 71) as determined by western blotting. (^*^p < 0.05, Student's *t*-test) (**E**) The relationship between miR-512-5p and JAG1 was performed by Pearson’s correlation test (r = -0.3352, represent inverse relationship). (**F**) JAG1 expression determined after transfecting cells with corresponding vectors by western blotting analysis. ^*^p < 0.05, ^**^p < 0.01, Student's *t*-test, compared to the Control group. Data are represented as the mean ± standard deviation of three independent experiments.

The bioinformatics databases (Targetscan, miRinda, and miRWalK) were used to predict the potential target genes of miR-512-5p. A Venn diagram depicts the overlap of commonly targeted miR-512-5p genes based on information from the databases (Figure 3A). Among them, JAG1 was identified as a potential gene of miR-512-5p. The target binding site of miR-512-5p in the JAG1 3'-UTR was predicted by Targetscan (Figure 3B) and confirmed by dual luciferase reporter gene assays, which showed that miR-512-5p overexpression inhibited luciferase activity of the wild type (WT) group in U87 cells, while luciferase activity of the mutant (MUT) group remained unchanged (Figure 3C). Conversely, miR-512-5p silencing increased luciferase activity of the wild type (WT) group in LN229 cells, while luciferase activity of the mutant (MUT) group remained unchanged (Figure 3C). Western blotting assays showed that JAG1 positively correlated with glioma metastasis (Figure 3D). We also verified the correlation of miR-512-5p and JAG1 expression in glioma tissues (*n* = 71) and NBTs (*n* = 8) by RT-qPCR and western blotting. Furthermore, the Spearman correlation test indicated that JAG1 expression was negatively correlated with miR-512-5p expression in glioma tissues (Figure 3E) (*r* = -0.3352 and *p* = 0.043, respectively). Moreover, western blotting assays further confirmed that miR-512-5p negatively regulated JAG1 expression in U87 and LN229 cells. These results imply that miR-512-5p overexpression decreases JAG1 expression in U87 cells, whereas miR-512-5p silencing increases JAG1 expression in LN229 cells (Figure 3F). Taken together, these findings suggest that JAG1 is a downstream target of miR-512-5p in GBM.

### JAG1 mediates the effect of miR-512-5p on GBM cells

**Figure 4:**
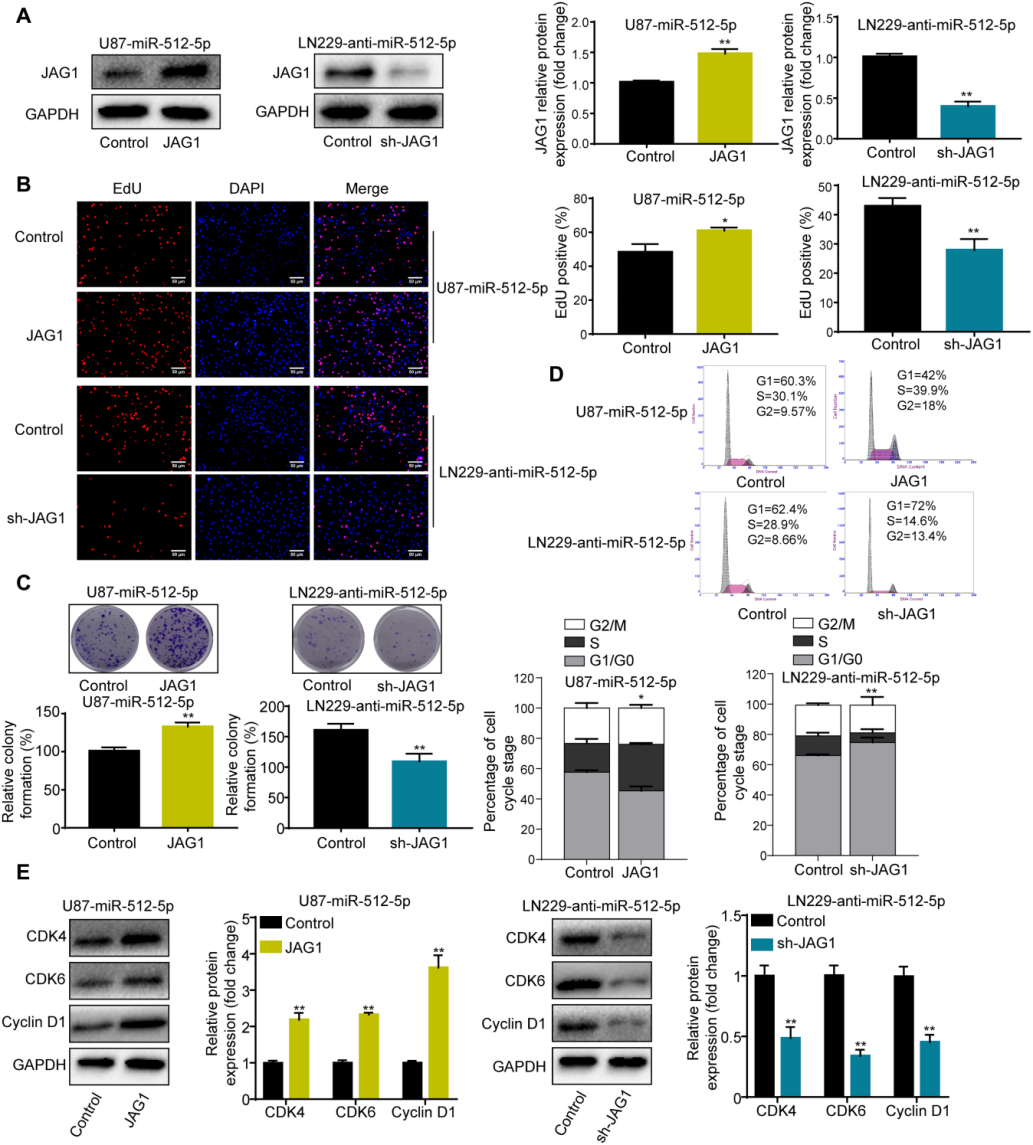
**JAG1 mediates the effect of miR-512-5p on GBM cells.** (**A**) JAG1 expression determined by western blotting after transfecting U87-miR-512-5p or LN229-anti-miR-512-5p cells with JAG1 or sh-JAG1. (**B**) Cell proliferation activity as examined by EdU assays. (200 ×) (**C**) Cell proliferation activity as examined by colony formation assays. (**D**) Analysis of cell cycle by flow cytometry. (**E**) G1-arrest-relevant cell cycle regulators determined by western blotting. Data are represented as the mean ± standard deviation of three independent experiments. ^*^p < 0.05, ^**^p < 0.01, Student's *t*-test, compared to the Control group.

To investigate whether JAG1 affects the role of miR-512-5p, we performed rescue experiments by transfecting JAG1 and short hairpin RNA (shRNA) in U87 and LN229 cells. JAG1 expression was restored after overexpressing JAG1 in U87-miR-512-5p cells (Figure 4A). JAG1 restoration rescued the inhibitory effect of miR-512-5p on cell proliferation and the G1-S phase cell cycle in U87-miR-512-5p cells (Figure 4B–4E). In contrast, silencing JAG1 enhanced the inhibitory effect of miR-512-5p on cell proliferation and the G1-S phase cell cycle in LN229-anti-miR-512-5p cells (Figure 4A–4E). These findings indicate that JAG1 is a functional downstream target of miR-512-5p in GBM.

### BMSC-exosomal miR-512-5p can be internalized by U87 cells

**Figure 5:**
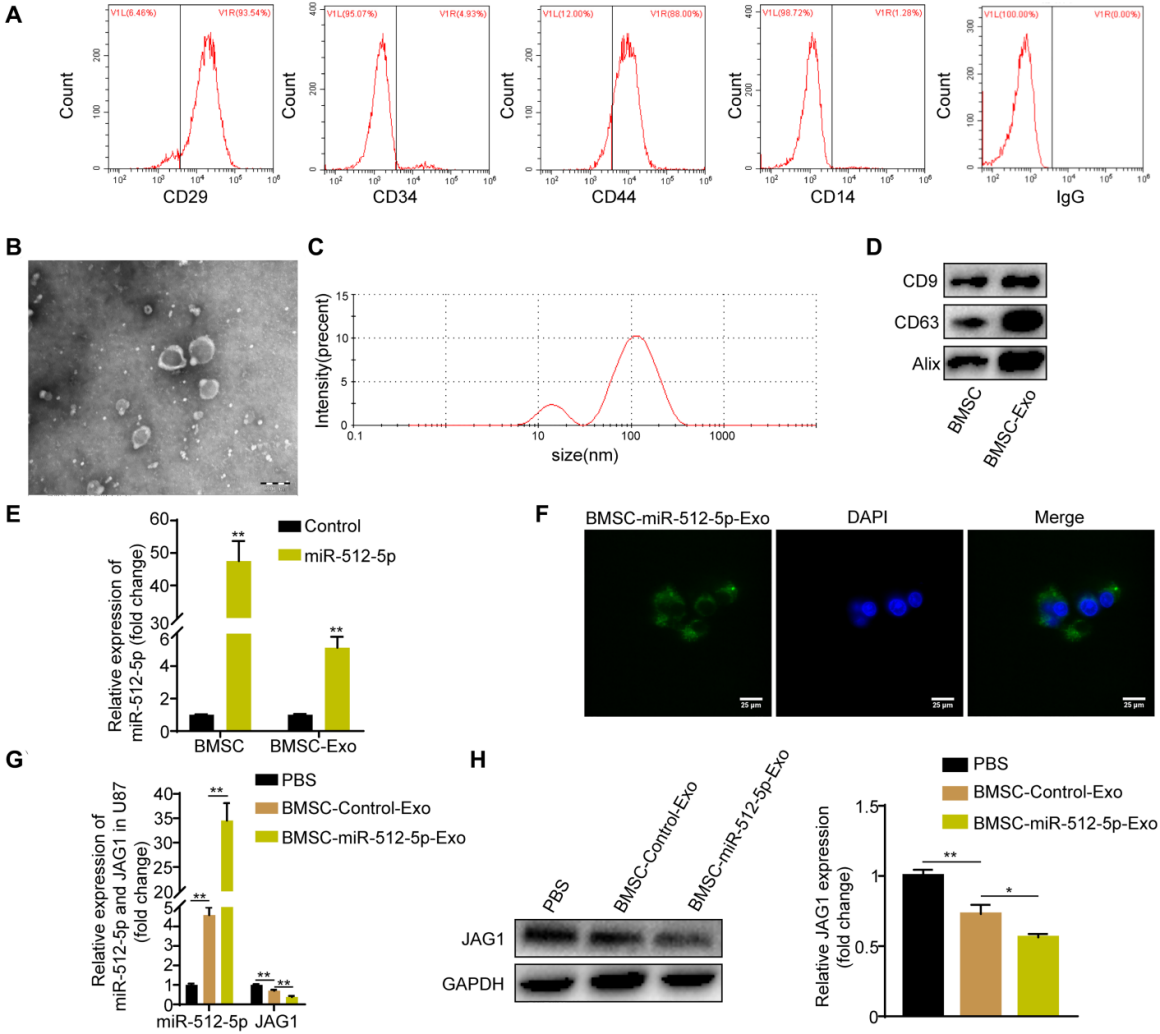
**BMSC-exosomal miR-512-5p can be internalized by U87 cells.** (**A**) Surface marker proteins of BMSC identified by flow cytometry assays. (**B**) Representative TEM image of BMSC exosomes. (scale bar, 200 μM) (**C)** Particle size distribution of BMSC exosomes as measured by DLS. (**D**) Exosome surface markers determined by western blotting. (**E**) miR-512-5p expression found in BMSC and BMSC-derived exosomes by RT-qPCR after transfection with miR-512-5p or the Control. (**F**) BMSC-exosomal miR-512-5p internalized by U87 cells. (scar bar = 25 μM). (**G**) miR-512-5p and JAG1 expression determined by RT-qPCR in U87 cells after co-culture with BMSC-derived exosomes or PBS. (**H**) JAG1 expression determined by western blotting in U87 cells after co-culture with BMSC-derived exosomes or PBS. Data are represented as the mean ± standard deviation of three independent experiments. ^*^p < 0.05, ^**^p < 0.01, Student's *t*-test, compared to the Control group.

CD44 and CD29 were highly expressed in BMSC while CD14 and CD34 were poorly expressed as determined by flow cytometry (Figure 5A), indicating successful isolation of BMSC. BMSC-derived exosomes were confirmed by TEM (Figure 5B) and DLS (Figure 5C) after ultracentrifugation. The extracted vesicles were spherical and cup-shaped with a diameter of 20–200 nm. Surface membrane proteins (CD63, CD9, Alix) were highly enriched in our isolated exosomes as shown by western blotting (Figure 5D). These results confirm that our isolated vesicles were exosomes. Notably, both BMSC and BMSC-derived exosomes had high miR-512-5p expression after miR-512-5p transfection (Figure 5E). Using fluorescence microscopy, we found that BMSC-derived exosomes could be internalized by U87 cells (Figure 5F). Exosomes from BMSCs transfected with miR-512-5p (BMSC-miR-512-5p-Exo) or controls (BMSC-Control-Exo) were incubated with U87 cells. This increased miR-512-5p expression but decreased JAG1 expression in BMSC-miR-512-5p-Exo based on RT-qPCR and western blotting assays (Figure 5G, 5H).

### BMSC-exosomal miR-512-5p inhibits GBM cell proliferation and causes cell cycle arrest

**Figure 6:**
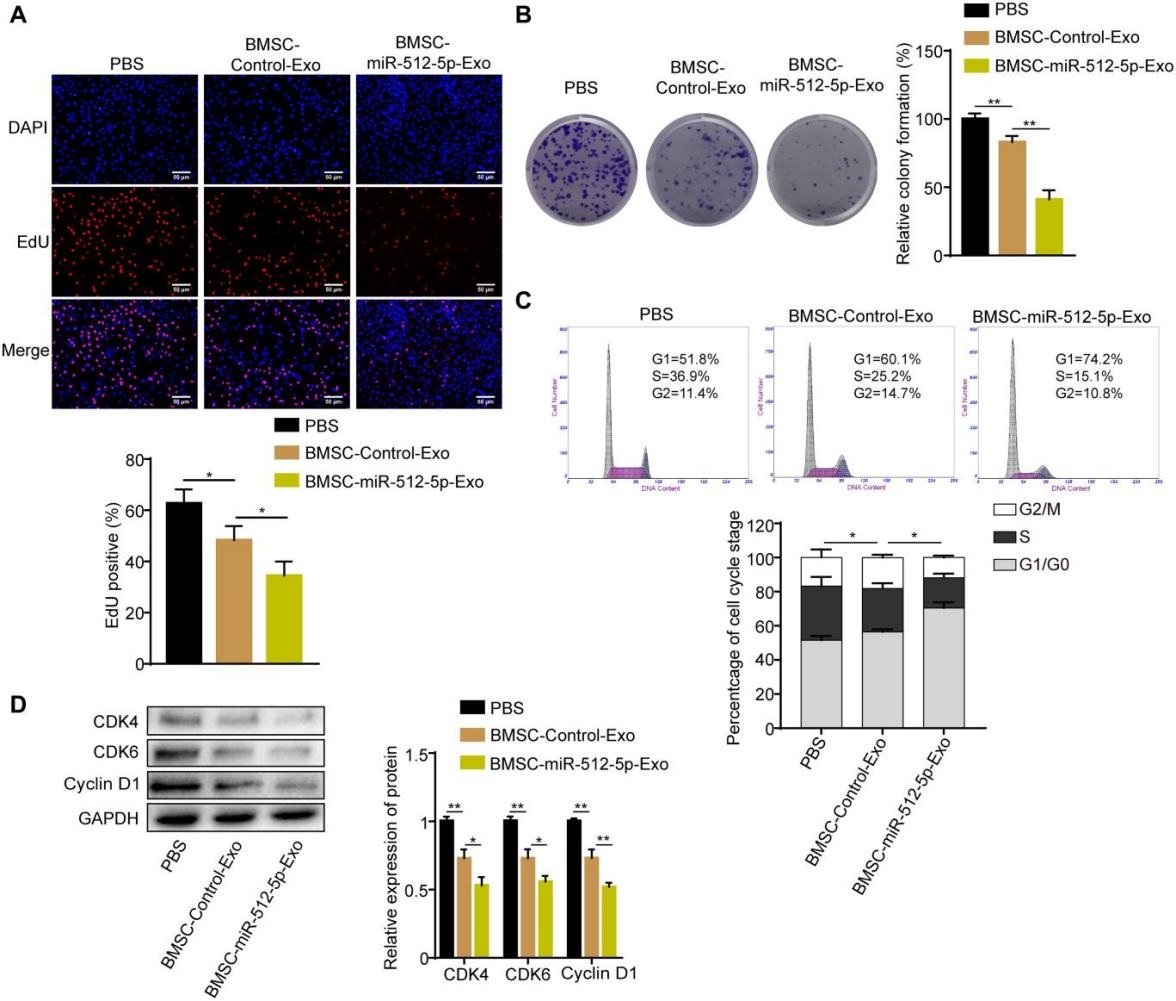
**BMSC-exosomal miR-512-5p inhibits GBM cell proliferation and causes cell cycle arrest.** (**A**) Cell proliferation activity as examined by EdU assays. (200 ×) (**B**) Cell proliferation activity as examined by colony formation assays. (**C**) Analysis of cell cycle by flow cytometry. (**D**) G1-arrest-relevant cell cycle regulators determined by western blotting assays. Data are represented as the mean ± standard deviation of three independent experiments. ^*^p < 0.05, ^**^p < 0.01, Student's *t*-test, compared to the Control group.

To further explore whether the exosomes derived from BMSC carrying miR-512-5p affects GBM cell function, U87 cells were co-cultured with BMSC-derived exosomes or PBS. U87 cells co-cultured with BMSC-derived exosomes (BMSC-miR-512-5p-Exo or BMSC-Control-Exo) had inhibited proliferation and G1-S phase cell cycle compared to the PBS group based on EdU, colony formation, and flow cytometry assays (Figure 6). In addition, treatment with BMSC-miR-512-5p-Exo reduced cell proliferation and induced cell cycle arrest to a greater extent than the BMSC-Control-Exo group. These data suggest that BMSCs can deliver miR-512-5p via exosomes to regulate GBM cell proliferation and cell cycle.

### BMSC-exosomal miR-512-5p inhibits tumor growth *in vivo*

**Figure 7:**
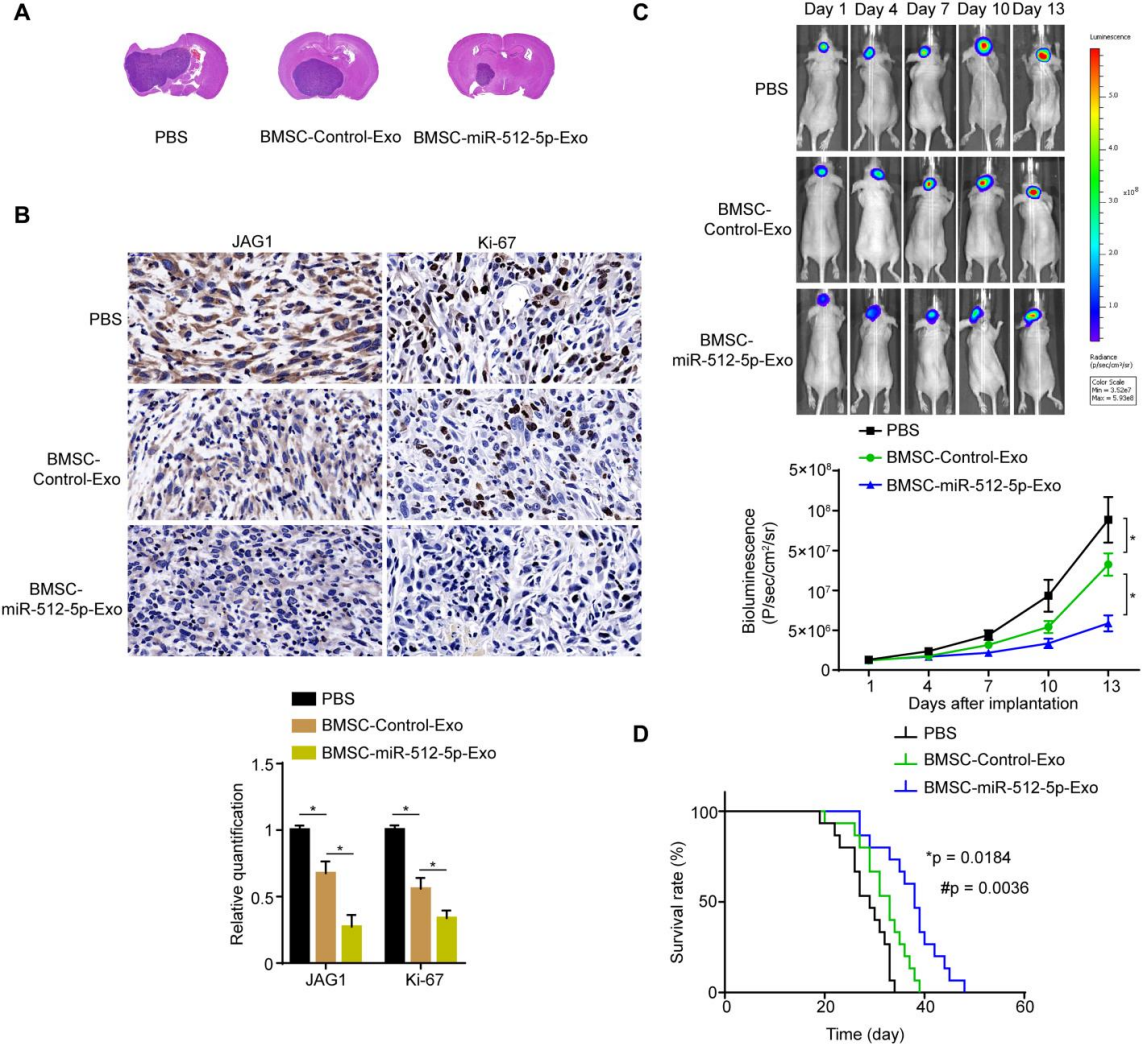
**BMSC-exosomal miR-512-5p inhibits tumor growth *in vivo*.** (**A**) Effect of BMSC-exosomal miR-512-5p as determined by HE in nude mice. (**B**) JAG1 and Ki-67 expression as determined by IHC. (scale bar = 50 μM) (**C**) Tumor growth as determined by bioluminescence imaging. after treatment with PBS or BMSC-derived exosomes. (**D**) Overall survival of nude mice receiving treatment was determined by Kaplan-Meier analysis. (*n* = 15, respectively). (^*^p = 0.0184, comparing the BMSC-Control-Exo group with the PBS group, ^#^*p* = 0.0036, comparing the BMSC-miR-512-5p-Exo group with the PBS and BMSC-Control-Exo groups, Log-Rank test).

Next, we injected BMSC-miR-512-5p-Exo, BMSC-Control-Exo, and PBS into nude mice through the tail vein. The whole-brain section H&E staining showed that the xenograft tumors of the BMSC-derived exosomes group were smaller than that of the PBS group. The xenograft tumors of the BMSC-miR-512-5p-Exo group was significantly smaller than the BMSC-Control-Exo group (Figure 7A). Furthermore, JAG1 and Ki-67 expression was lower in the BMSC-derived exosomes group relative to the PBS group by immunohistochemistry analysis, with expression for the BMSC-miR-512-5p-Exo group being significantly lower than the BMSC-Control-Exo group (Figure 7B). Moreover, bioluminescence imaging of the U87 GBM intracranial model showed that it grew more slowly in the BMSC-derived exosomes group compared to the PBS group, and the tumor growth of BMSC-miR-512-5p-Exo group was significantly slower than the BMSC-Control-Exo group (Figure 7C). The survival time of nude mice showed that the BMSC-miR-512-5p-Exo group significantly prolonged survival compared to the BMSC-Control-Exo and PBS groups (Figure 7D). In conclusion, these results suggest that the BMSC-exosomal miR-512-5p inhibits GBM growth *in vivo*.

## DISCUSSION

Despite aggressive multimodal treatments, the overall prognosis for GBM patients is poor [30]. Exosomes are important mediators in the crosstalk between GBM and the tumor microenvironment [31]. Mesenchymal stem cells are considered a foreground treatment due to their glioma homing properties and exosome production capacity [32, 33]. Recent evidence revealed that engineered MSC-derived exosomes regulate glioma development [34]. In this study, we found down-regulation of miR-512-5p may contribute to tumor progression, and further demonstrated that BMSC-derived exosomes delivering miR-512-5p could suppress GBM progression by negatively regulating JAG1. This highlights the potential for miR-512-5p to serve as a novel GBM therapeutic target.

Initially, we found that miR-512-5p expression was downregulated in glioma from the CGGA dataset. Similarly, Ciafrè et al. validated that the expression of multiple miRNAs was significantly altered in tumors with peripheral brain areas from the same patient [35]. We also found that miR-512-5p was downregulated in GBM samples and tissues. Overexpressed miR-512-5p inhibited cell proliferation and cell cycle G1-S phase transition. Poor miR-512-5p expression has been exhibited in solid tumors [36, 37]. Zhang et al. determined that upregulation of miR-512-5p contributes to the reduction of radio-resistance in cervical cancer cells by targeting MUC1 [38]. Yang et al. found that downregulation of miR-512-5p enhances cell proliferation and participates in circ-0004277-mediated colorectal cancer cell proliferation [39]. miR-512-5p was also able to augment cisplatin-induced apoptosis and inhibit cell migration in lung cancer cells by targeting TEAD4 [40]. These studies implicate miR-512-5p involvement in tumor progression, and when combined with our data, suggest that miR-512-5p may act as an important mediator of tumor progression.

JAG1 is the most classic Notch ligand, which releases soluble proteins and regulates the Notch signaling pathway by ADAM17-mediated proteolytic cleavage [41]. JAG1 upregulation was found in GBM [25], and JAG1 silencing inhibited glioma cell proliferation [26]. In our study, we predicted the targets of miR-512-5p by bioinformatic datasets and constructed the target binding site of miR-512-5p in the JAG1 3'-UTR. Furthermore, we found that JAG1 was the target of miR-512-5p through dual luciferase assays, and JAG1 was highly expressed in GBM. Knockdown of JAG1 inhibited cell proliferation and cell cycle G1-S phase transition, and restoration of JAG1 rescued the inhibitory effect of miR-512-5p in GBM cells. JAG1 downregulation can induce cell cycle arrest to the G0 \G1 phase by regulating the binding of Notch and cyclin D1 promoter in breast cancer cells [42]. Our results also showed that JAG1 silencing could decrease cyclin D1 expression. Moreover, cyclin D1 is a key mediator in cell cycle regulation by binding to cyclin-dependent kinase (CDK4, CDK6) [43, 44]. Taken together, these findings suggest that miR-512-5p suppresses G1-arrest-relevant cell cycle regulators to inhibit GBM progression by targeting JAG1.

Exosomes originate from inside the cell, and genetic material is encapsulated during the release process, resulting in the exosomes having some genetic background of the mother cell [45, 46]. MSC-derived exosomes have cell-free therapeutic potential due to their safety and low immunogenicity [47]. Exosomes, as the vehicles of miRNA participating in cell communication, have attracted interest based on their potential role for tumor treatment. For example, Zhang et al. reported that BMSC-derived exosomal miR-206 inhibited the proliferation, migration, and invasion of osteosarcoma cells and induced their apoptosis [48]. Additionally, Wang et al. found that BMSC-derived exosomal miR-34 resulted in inhibition of GBM cell proliferation, invasion, migration, and tumorigenesis *in vitro* and *in vivo* [28]. Consistent with these findings, we found that BMSC-derived exomosal miR-512-5p inhibits GBM cell proliferation and induces cell cycle arrest by regulating JAG1. Interestingly, Lang et al. found that MSCs can be used as a natural bio-factory to produce Exo-miR-124 for glioma treatment [49]. These studies have shown that encapsulating miRNA into exosomes has a better effect than simply using miRNA, probably because exosomes have better stability in the body. Our findings confirmed that miR-512-5p packaged into MSCs exosomes were internalized by U87 cells. We delivered BMSC-miR-512-5p-Exo to human GBM xenografts in mice by tail vein injection and found that BMSC-derived exosomal miR-512-5p inhibited GBM growth. There is evidence that exosomes containing miRNAs act as important mediators in tumor development [50, 51].

**Figure 8:**
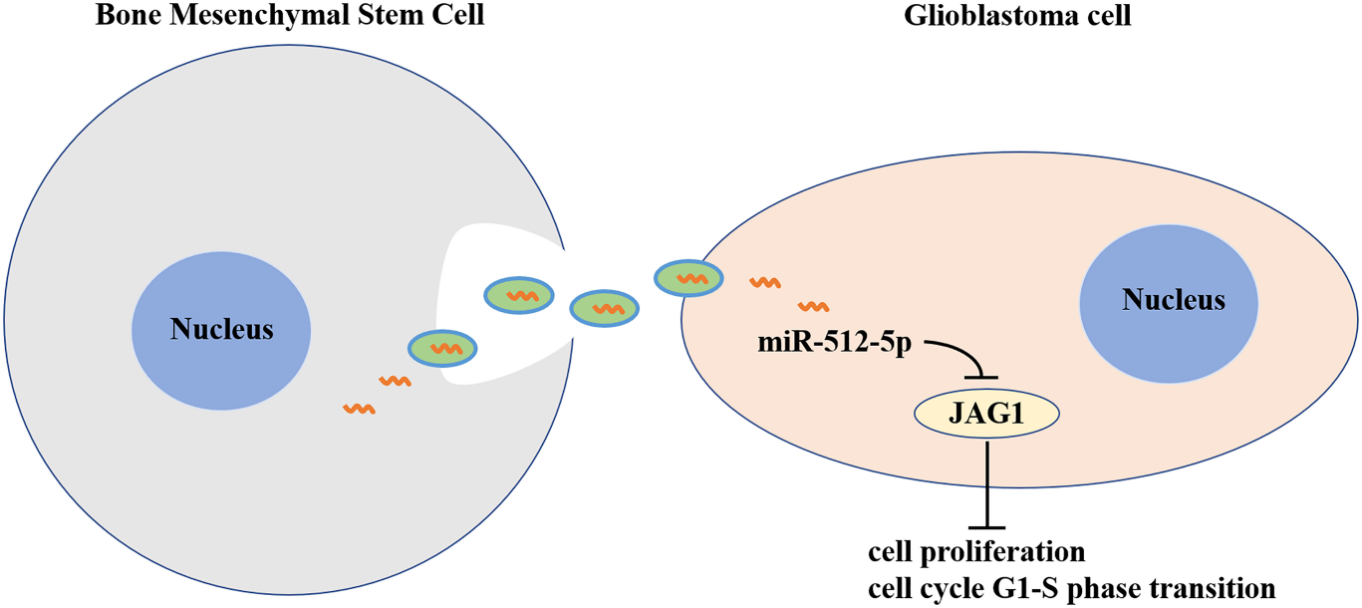
**Exosomes derived from MicroRNA-512-5p transfected bone mesenchymal stem cell inhibit glioblastoma progression by targeting JAG1.** BMSC-exosomal miR-512-5p inhibited the expression of JAG1 in GBM, and suppressed cell proliferation and G1-S phase cell cycle by inhibiting the expression of CDK4, CDK6 and Cyclin D1.

In this context, our study demonstrated that BMSC exosomes can deliver miR-512-5p into GBM cells and inhibit GBM progression by targeting JAG1 (Figure 8). These findings provide insight into the mechanism of molecular therapy for GBM treatment and highlights the potential for BMSC-Exo as a vehicle to transport miR-512-5p into GBM.

## MATERIALS AND METHODS

### Public datasets and human tissue samples

MiRNA expression microarray data were downloaded from the Chinese Glioma Genome Atlas (CGGA) database (http://www.cgga.org.cn/). The target genes of miR-512-5p were predicted by the TargetScan database (http://www.targetscan.org/ vert_71/), the miRwalk database (http://mirwalk.umm.uni-heidelberg.de/) and the miRanda database (http://www.miranda.org/).

This study was approved by the Ethics Committee of Nanchang University. Clinical samples were collected from the Department of Neurosurgery, Second Affiliated Hospital of Nanchang University. The glioma samples were collected from surgical specimens after obtaining consent from the patients (WHO I, *n* = 7; WHO II, *n* = 26; WHO III, *n* = 10; WHO IV = 28). Non-tumor brain tissues (NBTs, *n* = 8) were derived from the surgical specimens of patients with traumatic brain injury.

### Fluorescence *in situ* hybridization (FISH) and cell culture

The miR-512-5p probe 5′…DIG-GAAAGTGCCCTCAAGGCTGAGTG-DIG…3′ was designed and synthesized from Servicebio Technology (Wuhan, China). FISH analysis was performed as previously described [52].

Normal human astrocyte (NHA) cell line, and human GBM cell lines U87, T98, U251, LN229, and A173 were obtained from the Cell Bank of the Chinese Academy of Science (Shanghai, China), and were cultured in Dulbeccos modified eagle medium (DMEM) supplemented with 10% fetal bovine serum (FBS), and 1% penicillin /streptomycin. All cell lines were maintained at 37°C with 5% CO2.

Bone Mesenchymal stem cell (BMSC) obtained from healthy human bone marrow (BM), the isolation and culture of BMSC were performed as previously described [29]. The third generation of BMSC was isolated and cultured. Mouse anti-human antibodies against CD34, CD44, CD14, and CD29 were used to identify cell surface antigens by flow cytometry. CD44 and CD29 were used as markers of BMSC; CD14 and CD34 were used as markers for hematopoietic stem cells.

### Cell transfection

Lentivirus expressing miR-512-5p or anti-miR-512-5p was constructed by Genechem (Shanghai, China) using Ubi-MCS-SV40-EGFP-IRES-puromycin vector. Lentivirus expressing JAG1 or JAG1 shRNA was constructed by Genechem (Shanghai, China) using CMV-MCS-SV40-Neomycin vector. Briefly, U87, LN229 and BMSC were transfected miR-512-5p or anti-miR-512-5p with polybrene and enhanced infection solution (Genechem, China) to generate stable cell lines. These stable cell lines were then used to examine the effects of miR-512-5p overexpression. The cells were collected and treated after 48 h of transfection.

### Protein isolation and western blotting analysis

Total protein isolation and western blotting analysis were performed as described previously [53]. The cells were collected after transfection or co-culture. The antibodies were used included Cyclin D1 (Abcam, UK), Ki67 (Abcam, UK), CDK4 (Abcam, UK), CDK9 (Abcam, UK), CDK63 (Abcam, UK), Alix (Abcam, UK), CDK6 (Abcam, UK), GAPDH (Abcam, UK) and JAG1 (Abcam, UK). The secondary antibodies containing anti-rabbit horseradish peroxidase (HRP) and anti-mouse HRP were obtained from Santa Cruz Biotechnology (Santa Cruz, CA, USA).

### RNA extraction and qRT-PCR

Total RNA was prepared using Total RNA extraction kit (Invitrogen, USA). The expression level of miR-512-5p was determined using a stem-loop-specific primer method as previously described [54]. The cDNAs were amplified by RT-qPCR using SYBR Premix Ex Taq (Takara, USA). Primers were purchased from Ribobio (Guangzhou, China), and the fold changes in expression were calculated by relative quantification (2^−ΔΔCt^); U6 RNA was used as an endogenous control. The primer sequences were provided by Ribobio (Guangzhou, China) (Table 2).

**Table 2:** Primer sequences of the genes for RT-qPCR.

**Genes**	**Primer sequences (5′-3′)**
miR-512-5p	F: CACTCAGCCTTGAGGGC R: AACCAACCAACCACAACAAC
JAG1	F: GCCGCATCTCACAGCTA R: AAGGGGACACACAACCAG
U6	F: CGCTTCGGCAGCACATATACTAT R:CGCTTCACGAATTTGCGTGTCAAT
GAPDH	F: GAACGGGAAGCTCACTGG R: GCCTGCTTCACCACCTTCT

Abbreviations: RT-qPCR, reverse transcription quantitative polymerase chain reaction; F, forward; R, reverse; miR-512-5p, microRNA-512-5p; JAG1, Jagg1; GAPDH, glyceraldehyde-3-phosphate dehydrogenase.

### Cell proliferation assay and cell cycle assay

The GBM cells were seeded in 6-well plates. After 15 days, each dish was washed using PBS, fixed with paraformaldehyde (4%) for 30 min and then stained with crystal violet (0.1%). The 5-ethynyl-2-deoxyuridine (EdU) kit (Ribobio, China) was used to estimate proliferation of GBM cells. EdU assay was performed as previously described [29]. The GBM cells were cultured in 6-well plates for 24 h. The cell cycle was detected using Cell Cycle Staining Kit (Multi Sciences, Hangzhou, China) by flow cytometry, the protocol was previously described [55].

### Luciferase reporter assay

U87 and LN229 cells were seeded in 24-well plates for the luciferase assay. GBM cells were transfected with wild-type (WT) or mutant (MUT) plasmids of JAG1 using Lipofectamine 2000 reagent (Invitrogen, USA). After 48 h incubation, the Dual Luciferase Reporter Assay Kit (Promega) was used to detect luciferase levels of harvested cells.

### Exosome purification and characterization

To collect purified exosomes, BMSC was cultured in medium with 10% exosome-free FBS for 72 h. Then, the exosomes were extracted after 300 g/5 min, 2,000 g/10 min, 10,000 g/30 min, 100,000 g/70 min. Dynamic light scattering (DLS) analysis and transmission electron microscopy (TEM) was used to identify the size of purified exosomes, the protocol was previously described [56].

### Exosomes uptake

The exosomes of BMSC were labeled using PKH26 Fluorescent green Cell Linker Kit (sigma, USA). Exosomes were extracted as described above, and then exosomes were incubated with U87 cells in the serum-free medium. After 24 h, the treated cells were fixed and stained with DAPI. Confocal fluorescence microscope was used to visualize the cellular uptake.

### *In vivo* studies

All animal experiments were approved by the Experimental Animal Ethics Committee of Nanchang University. Four-week-old nude mice were purchased from the Shanghai Experimental Animal Center of the Chinese Academy of Sciences. The nude mice were randomly divided into 15 mice per group (total mice, *n* = 45). The intracranial GBM was established as previously described [57]. Then paraffin-embedded tissue sections were determined with haematoxylin–eosin (H&E) and Immunohistochemistry (IHC). Tumor growth was determined by bioluminescence imaging.

### Statistical analysis

The statistical analyses were performed with GraphPad Prism 7. Student’s *t*-test, or one-way analysis of variance (ANOVA) was used to compare the means difference of two groups or multiple groups, respectively. Kaplan–Meier analysis (log-rank test) was used to determine nude mice`s overall survival. *P* < 0.05 (^*^) were considered to be statistically significant, and the data were presented as the mean ± standard deviation (SD) in all results. Pearson's correlations analysis was used to analyse the correlation between expression level of miR-512-5p and JAG1 protein in glioma tissues.

### Data availability statement

The data that supports the findings of this study are available from the corresponding author upon reasonable request.

## References

[1] Dolecek TA, Propp JM, Stroup NE, Kruchko C. CBTRUS statistical report: primary brain and central nervous system tumors diagnosed in the United States in 2005-2009. Neuro Oncol. 2012 (Suppl 5); 14:v1–49. 10.1093/neuonc/nos218 23095881 PMC3480240

[2] Ostrom QT, Bauchet L, Davis FG, Deltour I, Fisher JL, Langer CE, Pekmezci M, Schwartzbaum JA, Turner MC, Walsh KM, Wrensch MR, Barnholtz-Sloan JS. The epidemiology of glioma in adults: a "state of the science" review. Neuro Oncol. 2014; 16:896–913. 10.1093/neuonc/nou087 24842956 PMC4057143

[3] Omuro A, DeAngelis LM. Glioblastoma and other malignant gliomas: a clinical review. JAMA. 2013; 310:1842–50. 10.1001/jama.2013.280319 24193082

[4] Corso CD, Bindra RS, Mehta MP. The role of radiation in treating glioblastoma: here to stay. J Neurooncol. 2017; 134:479–85. 10.1007/s11060-016-2348-x 28271281

[5] Ambros V. microRNAs: tiny regulators with great potential. Cell. 2001; 107:823–26. 10.1016/S0092-8674(01)00616-X 11779458

[6] Holubekova V, Mendelova A, Jasek K, Mersakova S, Zubor P, Lasabova Z. Epigenetic regulation by DNA methylation and miRNA molecules in cancer. Future Oncol. 2017; 13:2217–22. 10.2217/fon-2017-0363 28976205

[7] Berindan-Neagoe I, Monroig PC, Pasculli B, Calin GA. MicroRNAome genome: a treasure for cancer diagnosis and therapy. CA Cancer J Clin. 2014; 64:311–36. 10.3322/caac.21244 25104502 PMC4461198

[8] Luo H, Xu R, Chen B, Dong S, Zhou F, Yu T, Xu G, Zhang J, Wang Y, You Y. MicroRNA-940 inhibits glioma cells proliferation and cell cycle progression by targeting CKS1. Am J Transl Res. 2019; 11:4851–65. 31497204 PMC6731435

[9] Allahverdi A, Arefian E, Soleimani M, Ai J, Nahanmoghaddam N, Yousefi-Ahmadipour A, Ebrahimi-Barough S. MicroRNA-4731-5p delivered by AD-mesenchymal stem cells induces cell cycle arrest and apoptosis in glioblastoma. J Cell Physiol. 2020; 235:8167–75. 10.1002/jcp.29472 31957033

[10] Li Z, Zhang J, Zheng H, Li C, Xiong J, Wang W, Bao H, Jin H, Liang P. Modulating lncRNA SNHG15/CDK6/miR-627 circuit by palbociclib, overcomes temozolomide resistance and reduces M2-polarization of glioma associated microglia in glioblastoma multiforme. J Exp Clin Cancer Res. 2019; 38:380. 10.1186/s13046-019-1371-0 31462285 PMC6714301

[11] Dong ZQ, Guo ZY, Xie J. The lncRNA EGFR-AS1 is linked to migration, invasion and apoptosis in glioma cells by targeting miR-133b/RACK1. Biomed Pharmacother. 2019; 118:109292. 10.1016/j.biopha.2019.109292 31545240

[12] Fan H, Yuan R, Cheng S, Xiong K, Zhu X, Zhang Y. Overexpressed miR-183 promoted glioblastoma radioresistance via down-regulating LRIG1. Biomed Pharmacother. 2018; 97:1554–63. 10.1016/j.biopha.2017.11.050 29793318

[13] Hu Q, Liu F, Yan T, Wu M, Ye M, Shi G, Lv S, Zhu X. MicroRNA-576-3p inhibits the migration and proangiogenic abilities of hypoxia-treated glioma cells through hypoxia-inducible factor-1α. Int J Mol Med. 2019; 43:2387–97. 10.3892/ijmm.2019.4157 31017266 PMC6488173

[14] Ji CX, Fan YH, Xu F, Lv SG, Ye MH, Wu MJ, Zhu XG, Wu L. MicroRNA-375 inhibits glioma cell proliferation and migration by downregulating RWDD3 *in vitro*. Oncol Rep. 2018; 39:1825–34. 10.3892/or.2018.6261 29436665

[15] Xiao B, Zhou X, Ye M, Lv S, Wu M, Liao C, Han L, Kang C, Zhu X. MicroRNA‑566 modulates vascular endothelial growth factor by targeting Von Hippel‑Landau in human glioblastoma in vitro and in vivo. Mol Med Rep. 2016; 13:379–85. 10.3892/mmr.2015.4537 26572705

[16] Chu K, Gao G, Yang X, Ren S, Li Y, Wu H, Huang Y, Zhou C. MiR-512-5p induces apoptosis and inhibits glycolysis by targeting p21 in non-small cell lung cancer cells. Int J Oncol. 2016; 48:577–86. 10.3892/ijo.2015.3279 26648284

[17] Li J, Lei H, Xu Y, Tao ZZ. miR-512-5p suppresses tumor growth by targeting hTERT in telomerase positive head and neck squamous cell carcinoma *in vitro* and *in vivo*. PLoS One. 2015; 10:e0135265. 10.1371/journal.pone.0135265 26258591 PMC4530866

[18] Grochowski CM, Loomes KM, Spinner NB. Jagged1 (JAG1): Structure, expression, and disease associations. Gene. 2016; 576:381–84. 10.1016/j.gene.2015.10.065 26548814 PMC4673022

[19] Miele L, Golde T, Osborne B. Notch signaling in cancer. Curr Mol Med. 2006; 6:905–18. 10.2174/156652406779010830 17168741

[20] Reedijk M, Odorcic S, Chang L, Zhang H, Miller N, McCready DR, Lockwood G, Egan SE. High-level coexpression of JAG1 and NOTCH1 is observed in human breast cancer and is associated with poor overall survival. Cancer Res. 2005; 65:8530–37. 10.1158/0008-5472.CAN-05-1069 16166334

[21] Xiao HJ, Ji Q, Yang L, Li RT, Zhang C, Hou JM. *In vivo* and *in vitro* effects of microRNA-124 on human gastric cancer by targeting JAG1 through the Notch signaling pathway. J Cell Biochem. 2018; 119:2520–34. 10.1002/jcb.26413 28941308

[22] Chen J, Zhang H, Chen Y, Qiao G, Jiang W, Ni P, Liu X, Ma L. miR-598 inhibits metastasis in colorectal cancer by suppressing JAG1/Notch2 pathway stimulating EMT. Exp Cell Res. 2017; 352:104–12. 10.1016/j.yexcr.2017.01.022 28161537

[23] Liu X, Luo X, Wu Y, Xia D, Chen W, Fang Z, Deng J, Hao Y, Yang X, Zhang T, Zhou L, Wu Y, Wang Q, et al. MicroRNA-34a Attenuates Paclitaxel Resistance in Prostate Cancer Cells via Direct Suppression of JAG1/Notch1 Axis. Cell Physiol Biochem. 2018; 50:261–76. 10.1159/000494004 30282072

[24] Yi DY, Su Q, Zhang FC, Fu P, Zhang Q, Cen YC, Zhao HY, Xiang W. Effect of microRNA-128 on cisplatin resistance of glioma SHG-44 cells by targeting JAG1. J Cell Biochem. 2018; 119:3162–73. 10.1002/jcb.26469 29091297

[25] Jeon HM, Kim SH, Jin X, Park JB, Kim SH, Joshi K, Nakano I, Kim H. Crosstalk between glioma-initiating cells and endothelial cells drives tumor progression. Cancer Res. 2014; 74:4482–92. 10.1158/0008-5472.CAN-13-1597 24962027 PMC4295931

[26] Jubb AM, Browning L, Campo L, Turley H, Steers G, Thurston G, Harris AL, Ansorge O. Expression of vascular Notch ligands Delta-like 4 and Jagged-1 in glioblastoma. Histopathology. 2012; 60:740–47. 10.1111/j.1365-2559.2011.04138.x 22296176

[27] Barile L, Vassalli G. Exosomes: therapy delivery tools and biomarkers of diseases. Pharmacol Ther. 2017; 174:63–78. 10.1016/j.pharmthera.2017.02.020 28202367

[28] Wang B, Wu ZH, Lou PY, Chai C, Han SY, Ning JF, Li M. Human bone marrow-derived mesenchymal stem cell-secreted exosomes overexpressing microRNA-34a ameliorate glioblastoma development via down-regulating MYCN. Cell Oncol (Dordr). 2019; 42:783–99. 10.1007/s13402-019-00461-z 31332647 PMC12994354

[29] Yu L, Gui S, Liu Y, Qiu X, Zhang G, Zhang X, Pan J, Fan J, Qi S, Qiu B. Exosomes derived from microRNA-199a-overexpressing mesenchymal stem cells inhibit glioma progression by down-regulating AGAP2. Aging (Albany NY). 2019; 11:5300–18. 10.18632/aging.102092 31386624 PMC6710058

[30] Stupp R, Mason WP, van den Bent MJ, Weller M, Fisher B, Taphoorn MJ, Belanger K, Brandes AA, Marosi C, Bogdahn U, Curschmann J, Janzer RC, Ludwin SK, et al, and European Organisation for Research and Treatment of Cancer Brain Tumor and Radiotherapy Groups, and National Cancer Institute of Canada Clinical Trials Group. Radiotherapy plus concomitant and adjuvant temozolomide for glioblastoma. N Engl J Med. 2005; 352:987–996. 10.1056/NEJMoa043330 15758009

[31] Matarredona ER, Pastor AM. Extracellular Vesicle-Mediated Communication between the Glioblastoma and Its Microenvironment. Cells. 2019; 9:96. 10.3390/cells9010096 31906023 PMC7017035

[32] Xu F, Shi J, Yu B, Ni W, Wu X, Gu Z. Chemokines mediate mesenchymal stem cell migration toward gliomas *in vitro*. Oncol Rep. 2010; 23:1561–67. 10.3892/or_00000796 20428810

[33] Lamfers M, Idema S, van Milligen F, Schouten T, van der Valk P, Vandertop P, Dirven C, Noske D. Homing properties of adipose-derived stem cells to intracerebral glioma and the effects of adenovirus infection. Cancer Lett. 2009; 274:78–87. 10.1016/j.canlet.2008.08.035 18842332

[34] Xu H, Zhao G, Zhang Y, Jiang H, Wang W, Zhao D, Hong J, Yu H, Qi L. Mesenchymal stem cell-derived exosomal microRNA-133b suppresses glioma progression via Wnt/β-catenin signaling pathway by targeting EZH2. Stem Cell Res Ther. 2019; 10:381. 10.1186/s13287-019-1446-z 31842978 PMC6915914

[35] Ciafre SA, Galardi S, Mangiola A, Ferracin M, Liu CG, Sabatino G, Negrini M, Maira G, Croce CM, Farace MG. Extensive modulation of a set of microRNAs in primary glioblastoma. Biochem Biophys Res Commun. 2005; 334:1351–58. 10.1016/j.bbrc.2005.07.030 16039986

[36] Wang Z, Zhu X, Zhang T, Yao F. miR-512-5p suppresses the progression of non-small cell lung cancer by targeting β-catenin. Oncol Lett. 2020; 19:415–423. 10.3892/ol.2019.11102 31897154 PMC6923952

[37] Kolacinska A, Morawiec J, Fendler W, Malachowska B, Morawiec Z, Szemraj J, Pawlowska Z, Chowdhury D, Choi YE, Kubiak R, Pakula L, Zawlik I. Association of microRNAs and pathologic response to preoperative chemotherapy in triple negative breast cancer: preliminary report. Mol Biol Rep. 2014; 41:2851–57. 10.1007/s11033-014-3140-7 24469723 PMC4013446

[38] Zhang J, Wang L, Jiang J, Qiao Z. Elevation of microRNA-512-5p inhibits MUC1 to reduce radioresistance in cervical cancer. Cell Cycle. 2020; 19:652–65. 10.1080/15384101.2019.1711314 32126879 PMC7145329

[39] Yang L, Sun H, Liu X, Chen J, Tian Z, Xu J, Xiang B, Qin B. Circular RNA hsa_circ_0004277 contributes to malignant phenotype of colorectal cancer by sponging miR-512-5p to upregulate the expression of PTMA. J Cell Physiol. 2020. [Epub ahead of print]. 10.1002/jcp.29484 31960446

[40] Adi Harel S, Bossel Ben-Moshe N, Aylon Y, Bublik DR, Moskovits N, Toperoff G, Azaiza D, Biagoni F, Fuchs G, Wilder S, Hellman A, Blandino G, Domany E, Oren M. Reactivation of epigenetically silenced miR-512 and miR-373 sensitizes lung cancer cells to cisplatin and restricts tumor growth. Cell Death Differ. 2015; 22:1328–40. 10.1038/cdd.2014.221 25591738 PMC4495356

[41] Lu J, Ye X, Fan F, Xia L, Bhattacharya R, Bellister S, Tozzi F, Sceusi E, Zhou Y, Tachibana I, Maru DM, Hawke DH, Rak J, et al. Endothelial cells promote the colorectal cancer stem cell phenotype through a soluble form of Jagged-1. Cancer Cell. 2013; 23:171–85. 10.1016/j.ccr.2012.12.021 23375636 PMC3574187

[42] Cohen B, Shimizu M, Izrailit J, Ng NF, Buchman Y, Pan JG, Dering J, Reedijk M. Cyclin D1 is a direct target of JAG1-mediated Notch signaling in breast cancer. Breast Cancer Res Treat. 2010; 123:113–24. 10.1007/s10549-009-0621-9 19915977

[43] Qie S, Diehl JA. Cyclin D1, cancer progression, and opportunities in cancer treatment. J Mol Med (Berl). 2016; 94:1313–26. 10.1007/s00109-016-1475-3 27695879 PMC5145738

[44] Sherr CJ, Beach D, Shapiro GI. Targeting CDK4 and CDK6: From Discovery to Therapy. Cancer Discov. 2016; 6:353–67. 10.1158/2159-8290.CD-15-0894 26658964 PMC4821753

[45] Ge R, Tan E, Sharghi-Namini S, Asada HH. Exosomes in Cancer Microenvironment and Beyond: have we Overlooked these Extracellular Messengers? Cancer Microenviron. 2012; 5:323–32. 10.1007/s12307-012-0110-2 22585423 PMC3460057

[46] Mathivanan S, Simpson RJ. ExoCarta: A compendium of exosomal proteins and RNA. Proteomics. 2009; 9:4997–5000. 10.1002/pmic.200900351 19810033

[47] Timmers L, Lim SK, Arslan F, Armstrong JS, Hoefer IE, Doevendans PA, Piek JJ, El Oakley RM, Choo A, Lee CN, Pasterkamp G, de Kleijn DP. Reduction of myocardial infarct size by human mesenchymal stem cell conditioned medium. Stem Cell Res. 2007; 1:129–37. 10.1016/j.scr.2008.02.002 19383393

[48] Zhang H, Wang J, Ren T, Huang Y, Liang X, Yu Y, Wang W, Niu J, Guo W. Bone marrow mesenchymal stem cell-derived exosomal miR-206 inhibits osteosarcoma progression by targeting TRA2B. Cancer Lett. 2020; 490:54–65. 10.1016/j.canlet.2020.07.008 32682951

[49] Lang FM, Hossain A, Gumin J, Momin EN, Shimizu Y, Ledbetter D, Shahar T, Yamashita S, Parker Kerrigan B, Fueyo J, Sawaya R, Lang FF. Mesenchymal stem cells as natural biofactories for exosomes carrying miR-124a in the treatment of gliomas. Neuro Oncol. 2018; 20:380–90. 10.1093/neuonc/nox152 29016843 PMC5817945

[50] Katakowski M, Buller B, Zheng X, Lu Y, Rogers T, Osobamiro O, Shu W, Jiang F, Chopp M. Exosomes from marrow stromal cells expressing miR-146b inhibit glioma growth. Cancer Lett. 2013; 335:201–04. 10.1016/j.canlet.2013.02.019 23419525 PMC3665755

[51] Sharif S, Ghahremani MH, Soleimani M. Delivery of Exogenous miR-124 to Glioblastoma Multiform Cells by Wharton’s Jelly Mesenchymal Stem Cells Decreases Cell Proliferation and Migration, and Confers Chemosensitivity. Stem Cell Rev Rep. 2018; 14:236–246. 10.1007/s12015-017-9788-3 29185191

[52] Zhi T, Jiang K, Xu X, Yu T, Wu W, Nie E, Zhou X, Jin X, Zhang J, Wang Y, Liu N. MicroRNA-520d-5p inhibits human glioma cell proliferation and induces cell cycle arrest by directly targeting PTTG1. Am J Transl Res. 2017; 9:4872–87. 29218086 PMC5714772

[53] Yuan F, Liu B, Xu Y, Li Y, Sun Q, Xu P, Geng R, Den G, Yang J, Zhang S, Gao L, Liao J, Liu J, et al. TIPE3 is a regulator of cell apoptosis in glioblastoma. Cancer Lett. 2019; 446:1–14. 10.1016/j.canlet.2018.12.019 30639532

[54] Kramer MF. Stem-loop RT-qPCR for miRNAs. Curr Protoc Mol Biol. 2011; Chapter 15:Unit 15.10. 10.1002/0471142727.mb1510s95 21732315 PMC3152947

[55] Bychkov M, Shulepko M, Osmakov D, Andreev Y, Sudarikova A, Vasileva V, Pavlyukov MS, Latyshev YA, Potapov AA, Kirpichnikov M, Shenkarev ZO, Lyukmanova E. Mambalgin-2 Induces Cell Cycle Arrest and Apoptosis in Glioma Cells via Interaction with ASIC1a. Cancers (Basel). 2020; 12:1837. 10.3390/cancers12071837 32650495 PMC7408772

[56] Maroto R, Zhao Y, Jamaluddin M, Popov VL, Wang H, Kalubowilage M, Zhang Y, Luisi J, Sun H, Culbertson CT, Bossmann SH, Motamedi M, Brasier AR. Effects of storage temperature on airway exosome integrity for diagnostic and functional analyses. J Extracell Vesicles. 2017; 6:1359478. 10.1080/20013078.2017.1359478 28819550 PMC5556670

[57] Zeng AL, Yan W, Liu YW, Wang Z, Hu Q, Nie E, Zhou X, Li R, Wang XF, Jiang T, You YP. Tumour exosomes from cells harbouring PTPRZ1-MET fusion contribute to a malignant phenotype and temozolomide chemoresistance in glioblastoma. Oncogene. 2017; 36:5369–81. 10.1038/onc.2017.134 28504721 PMC5611480

